# Hybrid mechanistic–machine learning PK/PD models with digital biomarkers: from cage to clinic

**DOI:** 10.3389/fphar.2026.1815118

**Published:** 2026-07-06

**Authors:** Szczepan W. Baran, Stefano Gaburro

**Affiliations:** 1 Baran Cafe, Cambridge, MA, United States; 2 Tech4Pets.Healthcare, Cambridge, MA, United States; 3 Digital Preclinical Society (DiPS), Cambridge, MA, United States

**Keywords:** digital biomarkers, hybrid modeling, machine learning, mechanistic modeling, pharmacodynamics, pharmacokinetics, preclinical-to-clinical extrapolation, regulatory science

## Abstract

**Background:**

Pharmacometric PK/PD models remain central to dose selection for first-in-human studies, therapeutic drug monitoring, and animal-to-human extrapolation of exposure–response relationships, but modern datasets, including digital biomarkers from continuous monitoring, omics, and imaging, challenge traditional modeling assumptions. Hybrid mechanistic–machine learning (ML) approaches offer a structured way to combine causal pharmacological frameworks with data-driven flexibility.

**Methods:**

This review surveys the emerging landscape of hybrid mechanistic–ML PK/PD modeling with a focus on digital biomarker integration. We examine model architectures, covariate discovery methods, cross-species scaling strategies, and validation practices relevant to preclinical-to-clinical extrapolation. Literature was identified through PubMed, Scopus, and Web of Science using search terms combining pharmacokinetics, pharmacodynamics, machine learning, hybrid modeling, digital biomarkers, and cross-species pharmacology. Studies published between 2015 and 2025 were prioritized, with inclusion of foundational earlier work where necessary.

**Results:**

Hybrid approaches improve individual clearance estimation, covariate discovery, and cross-species PK scaling in defined settings, particularly when dense time-series data are available and the mechanistic model is structurally sound but parametrically under-identified. Integration of multimodal datasets introduces practical challenges around missingness, device drift, and data leakage that require explicit mitigation. Application examples span rodents, dogs, non-human primates, and minipigs, where continuous digital measures strengthen exposure–response inference.

**Discussion:**

The credibility of hybrid models depends on validation rigor, interpretability, and regulatory alignment rather than on algorithmic novelty. When mechanistic models fit well and sample sizes are small, adding an ML layer risks overfitting without measurable gain. Models that are prospectively tested, transparently documented, and fitted to a defined context of use will be more influential than those that are merely complex. We outline reporting and audit practices to support robustness, reproducibility, and regulatory review.

## 1 Introduction

Pharmacometric pharmacokinetics/pharmacodynamic (PK/PD) models remain central to dose selection for first-in-human studies, therapeutic drug monitoring, and animal-to-human extrapolation, but modern datasets are increasingly complex (Bai et al., 2019; Babrak et al., 2019; Aliferis and Simon, 2024; Kapoor and Narayanan, 2023; Obermeyer et al., 2019; Wynants et al., 2020; Linardatos et al., 2021; Jiménez-Luna et al., 2020). Digital biomarkers, omics, imaging, and high-frequency monitoring provide richer signals than traditional sparse sampling, yet they also introduce multivariate measurement noise across correlated data streams. Hybrid mechanistic–machine learning (ML) modeling offers a practical compromise: it preserves causal structure and physiological constraints while using ML for tasks such as feature extraction (i.e., the automated identification and construction of informative summary variables from raw, high-dimensional data streams such as continuous sensor recordings), nonlinear covariate effects, and residual pattern learning. The central question is where hybrid methods add measurable value, where they introduce risk, and what evidence is needed for credible use in drug development.

This review spans preclinical species (rodents, dogs, minipigs, non-human primates) and clinical settings, examining how digital measures collected in animal studies can inform exposure–response modeling that is carried forward into human dose selection and monitoring. The scope includes both preclinical digital biomarkers (e.g., home-cage behavioral monitoring, telemetry) and their clinical analogs (e.g., wearable sensors, remote patient monitoring).

In parallel, the pharmaceutical field is experiencing rapid growth in digital biomarkers and remote monitoring technologies that enable continuous measurement of physiological and behavioral endpoints. These digital PD anchors, activity levels from wearable sensors, telemetry data, or automated home-cage behavioral readouts in animal studies, provide longitudinal insight into drug effects that complement traditional sparse sampling of blood draws or clinic visits (Baran et al., 2022; Traynard, 2023). When appropriately qualified, such digital measures can serve as common endpoints between preclinical models and clinical trials that improve cross-species and cross-phase consistency (FDA, 2019; FDA, 2023a). Incorporating ML into population PK models has already shown tangible benefits. Hughes and Keizer (2021) demonstrated improved individual parameter estimation through ML-adapted Bayesian priors, and Destere et al. (2022) showed significant bias reduction in iohexol clearance estimation by layering ML error correction onto a population PK framework (Hughes and Keizer, 2021; Destere et al., 2022). These innovations in data collection and modeling set the stage for a systems pharmacology approach in which diverse data streams are merged into integrated models.

Regulatory science has started to accommodate these methods. The U.S. Food and Drug Administration (FDA) and other agencies have initiated frameworks for AI/ML in drug development, emphasizing rigorous validation, transparency, and context-specific credibility (FDA, 2023b; FDA, 2022). This review is written for pharmacometricians and DMPK scientists involved in population PK/PD modeling and cross-species scaling, with emphasis on practical integration of ML components and digital endpoints into established modeling workflows. We maintain a critical tone, addressing not only opportunities but also limitations, and focus on strategies that are scientifically defensible rather than speculative. Where sections present audit checklists, best practices, or illustrative scenarios, these reflect the authors’ synthesis and interpretation of the cited literature rather than direct reproduction of published protocols.

To set the stage, we first clarify key terminology in Box 1. We then address data wrangling for integrated PK/PD modeling, followed by a survey of hybrid model architectures. Next, we discuss covariate modeling and cross-species scaling, including case examples in large preclinical species. We examine common pitfalls with mitigation strategies. A summary section maps typical questions to recommended hybrid modeling techniques. Finally, the Discussion synthesizes findings, outlines future directions, and grounds expectations.

Box 1 **Key definitions and concepts.**

**Hybrid PK/PD modeling**

An approach that integrates mechanistic models with machine learning. The mechanistic component (e.g., a compartmental PK model or receptor-based PD model) encodes known biology and pharmacology. The ML component learns patterns from data, such as correcting model residuals or suggesting structural modifications. The goal is to retain interpretable parameters (e.g., clearance, volume, EC_50_) while using ML to improve fit or prediction where traditional models struggle (24; see also 13 for an applied example).

**Digital biomarker (Digital PD Anchor)**

An objective, quantifiable physiological or behavioral measure collected via digital devices (sensors, wearables, implantables, or automated systems) that serves as an indicator of drug response or disease state (term introduced in ref. 9). Examples include continuous heart-rate and activity monitoring in freely moving non-human primates via wearable sensor vests (Fletcher et al., 2012), detection of atrial fibrillation in large populations using sensor photoplethysmography (Perez et al., 2019), and automated tracking of rodent behavior in instrumented home-cage environments (Baran et al., 2022).

**Translational digital biomarker (TDB)**

A conceptual framework (defined in ref. 9) for an objective, quantifiable measure of a physiological or behavioral response to disease progression or therapy, captured via digital monitoring technologies, that is clinically relevant in both preclinical and clinical settings. A TDB can be measured in animals (e.g., in the home-cage) and in human patients using analogous digital sensors, enabling a directly translatable pharmacodynamic readout between laboratory and clinic (Baran et al., 2022) (see Section 7.5 for application).

**Indirect response model**

In pharmacodynamics, an indirect response model (also called a turnover model) describes drug effects that occur by modifying the production or loss of an endogenous mediator, rather than by an immediate direct action on the observed effect. The drug inhibits or stimulates the factors controlling either the input (synthesis) or dissipation (removal) of the response variable, producing a delayed onset and offset of effect (Dayneka et al., 1993). Indirect response models are distinguished from direct effect models by this mechanism and often exhibit hysteresis between drug concentration and effect.

**Tolerance model**

A pharmacodynamic model that incorporates mechanisms of diminishing response over time with continued drug exposure. Tolerance is characterized by decreased drug effect following repeated or prolonged administration, often due to adaptive changes such as receptor desensitization, downregulation, or physiological feedback that opposes the drug’s action. Mechanism-based tolerance models include features like feedback inhibition or precursor pool depletion to capture this waning of effect (Sharma et al., 1998).

**Mixture/latent class model**

In population PK/PD modeling, a mixture model assumes that the overall population comprises two or more subpopulations with different parameter values. Instead of forcing a single unimodal distribution, the model uses a combination of distributions to fit heterogeneous data (Kaila et al., 2007). This approach identifies latent subgroups, for example, “fast” and “slow” metabolizer classes, or responders versus non-responders, each with its own PK or PD parameters. By estimating class membership probabilities for each individual, mixture models can reveal hidden population structure and improve parameter estimates for diverse patient groups.

## 2 Data integration and wrangling for preclinical and clinical PK/PD

The data integration challenges described in this section apply across preclinical and clinical settings, though examples are drawn primarily from preclinical workflows where digital biomarker streams are most commonly paired with PK sampling.

Hybrid modeling depends on coherent integration of heterogeneous sources. Digital biomarkers can be continuous, high-frequency, and device-dependent, while PK sampling is often sparse and protocol-driven. Successful integration typically requires standardized time stamps, consistent units, and metadata describing device settings, housing conditions, and preprocessing steps. Feature construction should be aligned to the mechanistic question, for example, circadian phase, activity bouts, or temperature excursions over windows matched to exposure. Where endpoints differ in frequency, the model should represent the measurement process explicitly rather than forcing arbitrary resampling.

Treating these steps as part of the modeling pipeline is expected to improve reproducibility and reduce spurious lag or covariate effects, consistent with recommendations for preprocessing discipline in ML-based science (Kapoor and Narayanan, 2023; Zhang et al., 2022).

Sensor-enabled workflows also raise practical challenges around missingness and bias. Data gaps can arise from battery limits, signal dropout, animal handling, or cage changes. If missingness correlates with disease severity or treatment, naive imputation can bias exposure–response relationships. For instance, if sicker animals are handled more frequently for welfare checks, creating data gaps in continuous monitoring during those periods, imputing baseline-level activity during gaps would systematically underestimate the association between drug exposure and reduced locomotion in severely affected subjects. Sensor placement, algorithm updates, and site-specific husbandry can shift feature distributions between studies. Hybrid models should therefore include quality control flags, sensitivity analyses to preprocessing choices, and study- or site-level random effects where appropriate. External validation across independent datasets should be used where possible to demonstrate transportability.

Robust data quality control is essential. Hybrid modeling often aggregates data from multiple sources (different laboratories, sensor devices, clinical sites), leading to variability in data formats and quality. Manual data curation remains indispensable: raw data used for model training and validation must undergo meticulous checks to catch discrepancies and outliers (Baran, 2023). Automated anomaly detection can flag potential issues, but human-in-the-loop review is strongly recommended to verify and correct anomalies before modeling (Baran, 2023). If a wearable accelerometer records an implausibly high activity spike when a dog is actually sedated, perhaps due to a technical glitch, that artifact must be identified and addressed during data cleaning. Physiological sensor readings may drift over time, so periodic calibration data may need integration to adjust for sensor drift. Consistency of units and scales across data sources is a seemingly trivial but critical aspect: mixing up units (e.g., ng/mL versus μg/mL) or time bases (beats per minute versus per second) can derail a model. A structured data dictionary and standardized templates for all datasets help prevent such errors.

When integrating multimodal PK/PD data, the choice between simultaneous and sequential modeling matters. Simultaneous models fit PK and PD together, often improving coherence because exposure and effect parameters are estimated jointly and can borrow information from each other. This is useful when digital biomarkers provide dense timing information that informs effect delays or turnover. Sequential models fit PK first and then feed predicted concentrations into the PD model. They can be simpler and more stable when PK is well characterized, but they can under-propagate uncertainty if not handled carefully. Many workflows use a sequential approach early, then move to simultaneous models for key decisions or when the PD signal is tightly coupled to exposure.

A practical example of the integration challenge is home-cage behavioral monitoring paired with intermittent physiological sampling (Baran et al., 2022). Behavioral streams can be continuous and high-frequency, while blood pressure or glucose may be measured sparsely. Integration requires a shared timeline and explicit choices about resampling. Common solutions include down-sampling high-frequency signals into summary features over windows aligned to PK sampling, or interpolating sparse measures when physiologically justified. Time alignment, device metadata, and consistent units are essential in all cases, because small timing errors can create artificial lags that mislead PK/PD inference.

## 3 Hybrid modeling architectures

Having assembled high-quality integrated datasets, we turn to modeling strategies that combine mechanistic and ML elements.

To illustrate how ML components integrate into traditional ODE-based PK/PD models, consider a standard two-compartment PK model where the elimination rate is described by: dA/dt = -(CL/V) · A. In a hybrid formulation, the ML component may augment this as: dA/dt = -(CL/V) · A+ f_NN(covariates, t), where f_NN is a neural network that captures residual dynamics not explained by the mechanistic terms. The mechanistic parameters (CL, V) retain their pharmacological interpretation; the ML term absorbs systematic misspecification.

We describe three common categories, indirect response models, tolerance models, and mixture/latent-class models, along with algorithmic integrations such as error-correction models and neural-network augmentations of ordinary differential equations (ODEs).

### 3.1 Indirect response and delayed effects

Indirect response models (Types I–IV per Sharma and Jusko’s classification) handle drug effects that modify the production or loss of an endogenous mediator rather than acting immediately on the observed response (Dayneka et al., 1993; Sharma et al., 1998). These models capture lag times and accumulation but assume specific functional forms, often first-order kinetics for baseline turnover.

Hybrid enhancement applies when the delay or response pattern does not fit simple exponential dynamics. If the delay changes over time or with repeated dosing, or if there is a complex dependency on an unmodeled biomarker, an ML component can learn a data-driven function for part of the model. One demonstrated approach uses Neural Ordinary Differential Equations (Neural ODEs), where the ODE’s rate equations (the functions defining dC/dt) are partly governed by a neural network rather than a fixed parametric formula. Researchers have applied Neural ODE frameworks to PK/PD, allowing the model to learn dynamics that standard indirect-response models fail to capture (Lu et al., 2021). The model can enforce general principles (mass balance, non-negativity) while letting the ML component flexibly fit the shape of a delay or feedback function. However, such approaches need large datasets. Chou and Lin (2023) noted that data-hungry methods like deep neural networks may be impractical in early drug development where data are limited (Chou and Lin, 2023). A recommended practice is to start with a mechanistic indirect-response model and add ML complexity only when the data clearly warrant it.

### 3.2 Tolerance and feedback models

Tolerance, the diminished response to a drug with repeated or prolonged exposure [clinically illustrated, for example, by β2-agonist desensitization in asthma (Hancox et al., 2002)] is mechanistically modeled by introducing feedback loops or depletable mediators into PK/PD ODEs (Sharma et al., 1998).

In a hybrid context, ML can estimate or classify tolerance behavior at the individual level. Not all individuals develop tolerance at the same rate; patient-specific factors (pharmacogenomic or physiological) may govern this variability. A hybrid model could incorporate an ML sub-model that predicts tolerance model parameters, rate of feedback or precursor depletion, from individual-specific covariates or biomarker patterns. Data-driven algorithms can also help detect when tolerance is occurring: a change-point detection algorithm applied to the time-series of responses can flag when the slope of response versus time shifts from positive to negative (signaling tolerance onset). Once identified, a mechanistic model can be fit with those time segments in mind.

### 3.3 Mixture/latent-class models for heterogeneous responses

Mixture models address population heterogeneity that cannot be explained by known covariates, such as bimodal distributions of metabolizer phenotypes or responder/non-responder subpopulations (Kaila et al., 2007).

ML strengthens mixture modeling in two ways. First, clustering algorithms (unsupervised ML) can identify natural groupings in PK parameter estimates or individual response trajectories, indicating whether a mixture model is appropriate. Second, ML classification models can interpret the subpopulations *post hoc*: once a mixture model fits two classes, a decision tree or classifier can identify which covariates separate those classes. This generates mechanistic hypotheses. ML thus serves as a diagnostic adjunct that helps interpret latent classes in biologically meaningful terms. Kaila et al. (2007) demonstrated that mixture modeling effectively detects a non-responder subgroup, improving dose–response characterization. Published examples of fully integrated ML–mixture PK/PD modeling remain limited; current applications primarily use ML diagnostically (e.g., to detect bimodal distributions in residuals) rather than as a fully integrated estimation component. This represents an emerging application area.

### 3.4 Hybrid algorithmic integrations

Beyond structural patterns, several algorithmic hybrids have emerged. The error-correction model uses a traditional mechanistic model to generate an initial prediction, then layers an ML model to correct systematic error. Destere et al. (2022) provide an instructive example: they used a population PK model for Bayesian clearance estimation from sparse plasma samples, then trained XGBoost and LASSO regression models to predict and correct the bias in those Bayesian estimates (Destere et al., 2022). The result was a significant reduction in bias and improved precision of individual clearance estimates. The ML component functioned as an advanced error term, learning from data what biases the mechanistic method tends to exhibit and adjusting the output accordingly.

A related approach by Hughes and Keizer (2021) used ML to adapt the Bayesian prior in model-informed precision dosing. In their vancomycin study, a standard population PK model was used for Bayesian parameter estimation, but an ML algorithm learned to identify cases where the patient’s data were poorly described by the population prior and would benefit from a less informative prior. The ML classified when the standard prior was miscalibrated for an individual and suggested weakening it, leading to better predictive performance for drug levels (Hughes and Keizer, 2021). The pattern is general: ML assists parameter estimation or model selection rather than replacing the PK/PD model. The pharmacology remains in the mechanistic framework, but ML addresses places where rigid assumptions falter.

An emerging area is AI-driven mechanistic model structure search. Genetic algorithms or metaheuristic optimization can automatically explore different model forms, varying compartment numbers, presence of effect compartments, transit compartments, or feedback loops. These algorithms rapidly evaluate candidate model structures against data and converge on better hypotheses. Ismail et al. (2022) demonstrated a genetic algorithm-based tool that outperformed manual stepwise model selection in some scenarios (Ismail et al., 2022). However, Chou and Lin (2023) caution that the fitness function must be carefully designed to avoid bias toward overly simple models or those fitting dataset idiosyncrasies (Chou and Lin, 2023). Automated approaches still require expert oversight to ensure biological plausibility and interpretability. While promising, model structure search algorithms have yet to see widespread adoption in regulatory-facing work.

The unifying principle across hybrid patterns is that ML should play a supporting role, guiding, accelerating, or refining mechanistic models, not replacing them. Replacing mechanism-based models entirely with black-box ML undermines both extrapolation and insight generation (Chou and Lin, 2023). The following sections on covariate scaling and cross-species translation further illustrate how these hybrid patterns apply in real translational problems.

## 4 Covariate modeling and cross-species scaling

### 4.1 Traditional covariate modeling in PK/PD

A core task in pharmacometrics is explaining variability in PK/PD parameters through patient- or system-specific covariates. Population PK modeling quantifies how intrinsic factors (body weight, organ function, genetics) and extrinsic factors (diet, co-medications) contribute to inter-individual differences in drug concentrations (FDA, 2023b; FDA, 2022). The classic approach involves *a priori* selection of plausible covariates followed by stepwise regression (forward inclusion and backward elimination) to identify which factors significantly improve model fit. This process assumes relatively simple relationships, commonly linear or power-law, between covariates and PK parameters. For example, clearance might be modeled to increase with weight or decrease with age, reflecting known physiology. Such assumptions impose interpretability but can miss complex interactions. Covariate modeling is therefore a balancing act: it improves model precision and extrapolation by explaining variability, yet relies on the modeler’s hypotheses about which factors matter and how they influence PK/PD.

### 4.2 Allometric scaling and its limitations

In preclinical-to-clinical extrapolation, species can be treated as a covariate that must be bridged when moving from animals to humans. Allometric scaling relates clearance to body weight via a power law, commonly with an exponent near 0.75, while volume of distribution scales approximately linearly with body weight. This relationship reflects broad physiological regularities and often provides a reasonable first approximation across mammals. However, allometry fails when clearance is dominated by species-specific pathways, nonlinear binding, saturable transport, or enzyme differences. It can also be unreliable for biologics, where target-mediated drug disposition and FcRn recycling vary by species and tissue. Practical mitigation includes using multiple species when available, combining allometry with *in vitro* data such as microsomal stability or transporter assays, and moving to physiologically based pharmacokinetic (PBPK) models when mechanistic details are needed. Hybrid methods can further reduce bias by learning residual patterns in historical translation errors, but they must be constrained to prevent implausible extrapolation.

### 4.3 ML-guided covariate discovery

ML complements classical covariate modeling by learning nonlinear and high-dimensional associations that are difficult to specify manually. This is particularly useful as candidate covariates expand beyond demographics to include omics, imaging, and continuous digital biomarkers (see also Valderrama-Bahamóndez and Fröhlich, 2024). Rather than replacing the population model, ML is often most effective as a hypothesis generator: ranking features, detecting interactions, and suggesting functional forms. The proposed relationships should then be tested within a mechanistic or statistical PK/PD framework using standard diagnostics and uncertainty quantification.

Practical safeguards are needed. Overfitting is a risk when covariates outnumber subjects. Cross-validation should respect study design (e.g., leave-one-study-out or leave-one-site-out). Feature engineering and preprocessing should be locked before final model fitting to reduce leakage. When a covariate is adopted, it should be expressed in a way that supports clinical interpretation, such as a parameter shift over a clinically meaningful range, and verified to remain plausible under extrapolation to new populations.

### 4.4 Surrogate ML models for PK prediction

ML can also serve as a surrogate to predict PK parameters or exposure profiles. A common use case is early prediction of human clearance or volume from chemical descriptors and historical PK datasets. Kosugi and Hosea (2020) described a machine-learning approach that predicts human clearance from structure descriptors by using similarity to compounds with known PK (Kosugi and Hosea, 2020). Such *in silico* models are valuable for triage, prioritizing assays, and informing first-pass dosing assumptions when experimental data are sparse. However, surrogate ML models are sensitive to domain shift (i.e., a change in the statistical distribution of input data between the training and deployment datasets, such as differences in chemical series, patient population, or measurement technology): performance degrades for novel chemotypes, uncommon routes, or mechanisms under-represented in the training set. They also provide limited mechanistic insight into why a prediction is high or low. A pragmatic hybrid pattern is to use ML-predicted parameters as priors or initial conditions within mechanistic models, then update them as *in vitro* or animal data become available. This preserves speed early while keeping the translational workflow anchored to interpretable pharmacology.

### 4.5 ML-enhanced scaling strategies

Hybrid scaling combines mechanistic scaling with data-driven correction. One approach is ML-corrected allometry, where an ML model predicts when a compound is likely to deviate from standard allometric exponents based on its properties. The prediction adjusts the scaling relationship or introduces a covariate that captures the deviation. A related approach learns species-specific correction factors on top of PBPK or compartmental models, using historical translation errors as training signal. This is helpful when the mechanistic model captures broad physiology but systematic biases remain from unmodeled biology such as transporter expression or binding differences. The value depends on careful validation across chemotypes and species, and on transparent reporting of when the correction is expected to apply. Where possible, corrections should be linked to plausible mechanisms so the model remains interpretable and robust to extrapolation. Inauen et al. (2025) developed an augmented allometric scaling approach integrating ML with body-weight scaling to predict drug clearance in farm animals. This hybrid model improved accuracy modestly (about 61% of predictions within two-fold error versus approximately 51% with conventional allometry), illustrating both the potential and current limitations of ML augmentation of allometric scaling in large animals (Inauen et al., 2025).

### 4.6 Cross-species and intraspecies PD scaling

Scaling pharmacodynamics from animals to humans is typically harder than scaling PK. PK depends on distribution and clearance processes that often relate to size and physiology. PD reflects the drug–biology interaction, which can differ between species in target sequence, expression, pathway wiring, feedback control, and disease biology (Regan et al., 2025). A common starting point is the same structural model across species (e.g., Emax or indirect response), with key PD parameters allowed to vary by species and disease state. Parameters that may need re-estimation include potency metrics (EC_50_), system sensitivity (Emax), baseline turnover rates, and delay or tolerance terms. Where binding drives response, differences in receptor density or affinity shift apparent potency. Where downstream signaling dominates, similar binding can still yield different effect sizes due to species-specific feedback.

PD scaling usually benefits from explicit mechanistic anchors: receptor occupancy–effect relationships, biomarker link models, or pathway models that separate drug exposure from system response. Within species, covariates such as age, sex, disease severity, genotype, and concomitant therapy can create PD heterogeneity comparable to interspecies differences. Hybrid modeling helps by using mechanistic structure to constrain extrapolation while using data-driven components to learn how covariates shift potency or system parameters. The goal is rarely a single universal PD parameter set. It is a defensible mapping from exposure to response in the target human population, with uncertainty quantified for first-in-human and dose-ranging decisions.

### 4.7 Regulatory expectations for covariate justifications

Regardless of whether covariates are identified with traditional methods or ML, regulators expect covariate effects to be biologically plausible and clinically meaningful. In population PK/PD submissions, covariates support labeling decisions such as dose adjustment in renal or hepatic impairment, pediatrics, or drug–drug interactions. Sponsors should pre-specify plausible covariates where possible, describe the search strategy, and report how the final model balances fit, stability, and interpretability.

A defensible covariate rationale addresses three points. First, why the covariate could influence exposure or response mechanistically. Second, how large the effect is over the clinically observed range, including uncertainty and shrinkage. Third, whether the effect changes dosing, safety margins, or benefit–risk. For ML-suggested covariates, the same principles apply. ML can screen or rank features, but final inclusion should be supported by model diagnostics, sensitivity analyses, and clear clinical interpretation.

Transparent reporting of assumptions, missing data handling, and external validation or posterior predictive checks materially improve reviewability.

Covariate modeling and cross-species scaling aim to explain why exposure and response differ across individuals and species. Hybrid mechanistic–ML approaches can incorporate higher-dimensional covariates (including digital measures) while retaining the interpretability needed for translational use.

## 5 Translational case examples in large species

Large animal models, minipigs, dogs, and non-human primates, play a central role in later-stage preclinical studies due to their closer physiological similarity to humans. This section focuses on how hybrid mechanistic–ML approaches and digital measures apply in these species, emphasizing what each species uniquely contributes to translational PK/PD.

### 5.1 Minipig and pig models

The Göttingen minipig has gained traction as an alternative non-rodent species in pharmacology and toxicology. Pigs share key anatomical and physiological traits with humans: comparable cardiovascular anatomy, similar dermal structure, and analogous drug-metabolizing enzymes (Ellegaard Göttingen Minipigs, 2021; Dalgaard, 2015). These similarities make minipigs particularly valuable for dermal and subcutaneous drug absorption, where their skin thickness and fat content better approximate human conditions than small furred animals (Ellegaard Göttingen Minipigs, 2021).

For biologic drugs, minipigs show translational promise. Zheng et al. (2012) examined the pharmacokinetics of eight therapeutic IgG antibodies in Göttingen minipigs, finding that plasma clearance scaled nearly linearly with body weight (allometric exponent ∼0.98) and was highly predictive of human clearance in the absence of target-mediated disposition (Zheng et al., 2012). Minipig subcutaneous bioavailability approximated human values as well as or better than cynomolgus monkey, likely owing to human-like skin and lymphatic architecture (Zheng et al., 2012). The minipig neonatal Fc receptor (FcRn) also shows binding affinity for IgG comparable to humans and cynomolgus monkeys, supporting similar IgG recycling dynamics (Zheng et al., 2012).

Hybrid strategies can use parallel PBPK models for minipig and human, with an ML component trained on existing antibody datasets to inform parameters that are difficult to scale directly, such as tissue FcRn-related recycling or target-driven clearance terms. The anatomical model captures known physiological differences; the data-driven component addresses residual biases after mechanistic scaling. From a digital measures perspective, activity collar or jacket accelerometers and implantable telemetry in pigs continuously monitor heart rate, core body temperature, blood pressure, and locomotor activity. Telemetry studies in conscious minipigs show clear circadian patterns analogous to humans (Regan et al., 2025). Early work has validated that telemetry minipigs are a viable alternative to dogs for cardiovascular safety pharmacology, with high-quality ECG signals and reliable hemodynamic measurements. These digital biomarker streams can be integrated with drug exposure data, linking dense physiological time-series to PK through mechanistic exposure–response models.

### 5.2 Dog models

Dogs are a standard species for cardiovascular safety pharmacology, where telemetry enables continuous ECG, blood pressure, and activity recording. Wireless implant systems and jacket-mounted sensors provide high-frequency recording of QT-related endpoints and autonomic measures over relevant exposure windows. These datasets support PK/PD modeling of concentration–effect relationships, including hysteresis and tolerance. Dogs can be a useful non-rodent species for translational PK because some physiological and metabolic features may better approximate humans than rodents for specific pathways. However, important differences must be accounted for. Dogs exhibit an essentially absent cytosolic arylamine N-acetyltransferase (NAT) pathway and therefore cannot N-acetylate certain arylamine or hydrazine substrates. Canine gastrointestinal physiology and luminal conditions also differ from humans, which can alter oral absorption and formulation performance. These factors should be considered when using canine data to predict human PK. Ethical considerations and cost limit cohort sizes, so models should quantify uncertainty and guard against overfitting, particularly when adding ML components.

Hybrid approaches are most compelling in dogs when they link dense telemetry to exposure and mechanistic hypotheses. Examples include modeling QT liability with activity and circadian covariates, or linking hemodynamic signatures to receptor engagement. ML can extract stable features from noisy ambulatory traces, but the final exposure–response relationship should remain interpretable and simulation-ready. Published examples of full hybrid mechanistic–ML PK/PD integration in dogs remain limited, but the infrastructure (dense telemetry data, established concentration–QT modeling frameworks) is well suited for this approach. Clear definition of context of use, prespecified validation, and transparency about preprocessing are needed to support translation into human risk assessment.

### 5.3 Non-human primate models

Non-human primates (NHPs), particularly rhesus and cynomolgus monkeys, are often regarded as the most translationally relevant non-rodent species for certain physiological and immunological endpoints. They share high genetic homology, similar anatomy, and analogous organ function, which is especially relevant for complex biological systems like the immune system. NHPs are the species of choice for developing biologics that do not cross-react with lower species’ targets, and for therapeutic areas like neuroscience where rodents cannot recapitulate higher brain functions. Monoclonal antibodies and other large biologics are frequently tested in cynomolgus monkeys, as these primates often show the most human-like pharmacokinetics and immune responses (e.g., human IgG interacts with monkey FcRn and immune cells similarly to humans). NHP models of neurological diseases, such as MPTP-treated parkinsonian monkeys, are similarly valuable because primate brain structure and motor control parallel human function in ways rodents cannot fully recapitulate.

NHP use is limited by cost and ethics, so studies are typically small. When available, NHP data strongly influence first-in-human dose selection, especially for biologics. A frequent approach is single-species scaling from monkey to human, treating the NHP as closest surrogate, sometimes with adjustments for target expression or binding differences. Hybrid modeling can extract more information from small NHP datasets by integrating dense within-subject longitudinal PK/PD time-series data with mechanistic model structure and by formally quantifying uncertainty for extrapolation.

Digital monitoring is expanding in NHP studies. Wearables, remote sensors, and video analytics allow continuous tracking of behavior and physiology in group-housed settings, revealing drug effects missed by periodic observation. Examples include automated quantification of activity, sleep-like states, social interaction, and motor signatures indicating CNS side effects. Fletcher et al. (2012) reported early multi-sensor systems for remote monitoring in primates, demonstrating the feasibility of collecting high-resolution streams suitable for modeling (Fletcher et al., 2012). These data can be linked to PK through exposure–response models, or used as intermediate biomarkers bridging preclinical and clinical endpoints. Given the small sample sizes typical of NHP studies, the mechanistic core remains important for borrowing strength across time and between endpoints, while ML components assist with feature extraction from complex sensor signals.

#### 5.3.1 Multi-species integration

Consider a monoclonal antibody with both minipig and monkey data. The minipig may accurately reflect nonspecific clearance via FcRn-mediated recycling (linear clearance), while the monkey is necessary if target biology differs (e.g., the target is expressed in monkey but not pig). A hybrid modeling solution incorporates a target-mediated drug disposition (TMDD) component, with species-specific target expression levels. The minipig data anchor baseline clearance and volume parameters; monkey data (or human *in vitro* binding data) parametrize target binding and internalization. The integrated model simulates human PK by combining these elements, using each species for its particular strength. An ML component can be layered on this scaffold to learn cross-species correction terms or nonlinear covariate effects (for example, using historical antibody datasets to inform priors on poorly identified parameters), while the TMDD equations preserve interpretability for human simulation. This multi-species hybrid approach can be more predictive than either species alone.

## 6 Pitfalls in hybrid modeling and mitigation strategies

Hybrid mechanistic–ML PK/PD modeling with digital measures extends classical pharmacometrics, but it also introduces failure modes that are easy to miss in small, high-dimensional, time-series datasets. The most common pitfalls are overfitting, data leakage, device drift, limited generalizability, and interpretability gaps. Table 1 summarizes what goes wrong, how it presents, and the minimum mitigations and audit checks that should be documented for translational use. To make these expectations actionable, we provide a reusable audit checklist (Box 2) that can be applied as a pre-analysis and pre-decision gate. Box 2 includes a definition of out-of-distribution (OOD) detection, i.e., methods that flag when new input data fall outside the range of the training data and predictions may be unreliable.

**TABLE 1:** Common pitfalls in hybrid mechanistic–ML PK/PD modeling with digital measures and recommended mitigations.

Pitfall	What goes wrong	Warning signs	Mitigation strategies	Minimum audit checks
Overfitting	ML layer captures noise or spurious correlations when feature space exceeds sample size	Large train–validation gap; unstable cross-validation; hyperparameter sensitivity; external validation failure	Mechanistic constraints as structural regularizers; shrinkage or Bayesian priors; early stopping; nested cross-validation; preference for parsimonious models	Independent test set or prospective validation; learning curves; pre-specified model selection criteria; documented hyperparameter tuning
Data Leakage	Information from test subjects contaminates training through preprocessing, feature selection, or tuning	Implausibly high performance; collapse under strict re-split; inconsistency in prospective use	Split before preprocessing; fit transforms within training folds only; nested CV; subject-level and time-aware splits; strict data lineage control	Pipeline audit; sealed hold-out re-run; verification of fold isolation; explicit documentation of data splits
Device Drift	Sensor calibration or firmware changes create distribution shifts misinterpreted as biological signal	Baseline shifts tied to time, batch, or site; increased artifacts; instability across firmware versions	Routine calibration; drift detection algorithms; batch/time robustness checks; artifact filtering; monitoring of device metadata	Calibration logs; control charts; stress testing by batch and time; robustness analysis under missingness and noise
Bias and Limited Generalizability	Training data do not represent intended-use population or site variability	Subgroup performance disparities; external validation drop; inconsistent mechanistic parameter estimates	Diverse training cohorts; multi-site validation; mechanistic encoding of known covariates; cautious domain adaptation	Subgroup performance reporting; external cohort testing; sensitivity analyses; explicit intended-use statement
Interpretability Gap	Black-box ML obscures drivers, reduces trust, and complicates regulatory review	Inability to explain predictions; reliance on biologically implausible features; unstable feature importance	Prefer interpretable ML when feasible; use explainability tools diagnostically; preserve transparent mechanistic layer; parameter plausibility checks	Model card; documented feature drivers; biological plausibility review; reproducible code and parameter archive

Box 2 **Audit checklist for hybrid mechanistic–ML PK/PD with digital measures.**

Context of use is explicitly defined: intended decision, population, setting, and acceptable risk.

Data provenance is documented: sensor type, firmware, calibration, sampling, missingness, drift checks.

Preprocessing is locked: feature definitions, filtering, imputation, and unit harmonization are versioned.

Leakage controls are proven: subject-level splits, temporal splits, and no look-ahead from outcomes.

Mechanistic plausibility checks: parameter bounds, identifiability, and sensitivity analyses are recorded.

ML model governance: training code, hyperparameters, random seeds, and model card are archived.

Validation is staged: internal, external, and prospective performance with predefined success criteria.

Uncertainty and failure modes are communicated: confidence intervals, OOD detection, and guardrails.

Traceability for audit: data lineage, change control, access controls, and reproducible pipelines.

## 7 Mapping translational questions to hybrid modeling strategies

This section maps common translational drug development questions to recommended hybrid mechanistic–ML approaches.

### 7.1 Predicting human PK from preclinical data (first-in-human dose selection)

Traditional allometric scaling or physiologically-based PK simulations using animal data are combined with an ML model that adjusts for compound-specific biases. Umemori et al. (2025) developed a combined allometry and random forest method that improved human clearance predictions for first-in-human dose setting (Umemori et al., 2025). The ML component, a regression using *in vitro* clearance, lipophilicity, and related descriptors, learns systematic compound-specific deviations from standard allometric predictions, for example, compounds with unusually high protein binding or active transport that cause clearance to deviate from body-weight scaling expectations. The mechanistic scaffold provides the physiological framework; ML captures the chemical-specific residuals. For biologics, Patidar et al. (2025) demonstrated an mPBPK‐ML framework for early target pharmacology assessment, using virtual antibody candidates and target characteristics to predict target occupancy and inform design rules.

### 7.2 Enhancing individual PK estimates with sparse samples (therapeutic drug monitoring)

A population PK model provides Bayesian estimation of individual parameters from limited drug levels. An ML model then predicts and corrects the residual estimation error. Hughes and Keizer (2021) demonstrated that ML-guided adaptation of Bayesian priors reduced PK prediction errors by 12%–22% in vancomycin monitoring (Hughes and Keizer, 2021). Separately, ML models trained on simulation-rich data have been shown to infer antibiotic exposure (AUC) from just two samples, outperforming conventional MAP-based methods (Codde et al., 2024). The mechanistic model provides the pharmacological prior; ML adjusts for estimation biases arising from limited sampling or unusual patient covariates.

### 7.3 Quantifying tolerance development under chronic dosing

Mechanistic tolerance models incorporate feedback inhibition or mediator depletion into PK/PD ODEs, capturing the characteristic decline in effect over time (Sharma et al., 1998). The hybrid extension uses ML to predict individual tolerance parameters, rate of feedback, extent of precursor depletion, from baseline features such as genetic polymorphisms or digital biomarker patterns. The mechanistic structure governs the overall tolerance dynamics (delay and magnitude of effect decline); the ML sub-model explains why some individuals tolerate a drug faster than others. This approach keeps the model anchored in desensitization and downregulation pharmacology while gaining flexibility for personalized prediction.

### 7.4 Handling responder versus non-responder populations

A latent mixture PK/PD model estimates subpopulations with distinct drug response, for example, a fraction with no therapeutic response and another with normal sensitivity. ML classification is then used to predict class membership from PK exposure metrics, baseline laboratory values, and digital biomarker signatures within the population PK/PD framework. The mechanistic component estimates the magnitude of benefit in each subpopulation; the ML component enables prospective patient stratification.

### 7.5 Integrating continuous digital biomarkers with clinical endpoints

This is the setting where translational digital biomarkers (TDBs, as defined in Box 1) provide a framework for cross-species endpoint alignment. A joint longitudinal model posits an unobserved disease state trajectory that drives both the digital biomarker and the clinical endpoint simultaneously. Drug exposure is linked to the latent disease progression via a mechanistic PK/PD model. An ML component maps the latent state to noisy digital sensor output, functioning as a state-space model or learned measurement function. The clinical endpoint (e.g., a physician-derived score) is modeled as another manifestation of the same latent state. Digital measures are proxies, not direct readouts of biology. Their modeling value depends on making this proxy relationship explicit and testable. The clinical endpoint anchors interpretation; the digital stream provides temporal resolution. Neither replaces the other. This approach has been applied in autoimmune diseases to link continuous activity monitor outputs with discrete clinical scores (Romijnders et al., 2025).

### 7.6 Optimizing dose and regimen for target effect

A mechanistic PK/PD or quantitative systems pharmacology (QSP) model acts as a virtual patient environment, and an AI optimization algorithm (genetic algorithm, reinforcement learning) explores dosing policies that maximize efficacy while respecting safety constraints. De Carlo et al. (2024) embedded a population PK/PD cancer model into a Q-learning reinforcement learning framework, demonstrating that the AI-optimized regimen outperformed the standard protocol by over 10% on efficacy and safety metrics in simulation (De Carlo et al., 2024). The mechanistic model evaluates biological outcomes; the AI efficiently searches the space of possible dose regimens, including adaptive or pulsatile schedules that manual approaches would not explore.

These strategies share a common architecture: the mechanistic model provides a transparent, biology-based scaffold, and ML contributes targeted flexibility. The cited examples demonstrate that hybrid models produce measurably better predictive performance than either method alone in defined, validated settings.

## 8 Discussion and future outlook

### 8.1 Value of hybrid models

The case for hybrid models rests on a specific claim: that mechanistic PK/PD structure and ML flexibility are not just compatible but mutually corrective. Mechanistic frameworks constrain extrapolation where data are sparse. ML components absorb residual complexity that fixed functional forms miss. The evidence reviewed here supports this claim in two well-defined scenarios: when dense time-series data are available to train the ML layer, and when the mechanistic model is structurally correct but parametrically under-identified. Chakravarty et al. (2021) demonstrated that an AI-driven PK model could resolve identifiability challenges in drug distribution by optimizing unknown tissue partition parameters alongside a physiologically-based framework (Chakravarty et al., 2021). Walter et al. (2025) showed that ML–mechanistic integration can predict full plasma concentration–time profiles for over 1000 compounds in rats with accuracy comparable to purely data-driven models, while additionally enabling *in silico* experiments that would be impractical *in vivo* (Walter et al., 2025). In some cases, ML inputs reduced variability in predicted profiles by smoothing experimental noise (Walter et al., 2025). Outside the scenarios above, the added complexity of hybridization showed diminishing or undemonstrated returns. This distinction matters for deployment decisions.

When mechanistic models fit well, covariates are few and well characterized, and sample sizes are small, adding an ML layer risks overfitting without measurable gain. In early-phase studies with fewer than 20–30 subjects and sparse sampling, standard population PK models with allometric scaling remain the appropriate default. Hybridization should be justified by evidence of systematic model misspecification, not adopted as a general-purpose enhancement.

### 8.2 Alignment with quantitative systems pharmacology (QSP)

Hybrid PK/PD models interface naturally with quantitative systems pharmacology when mechanistic pathways are represented explicitly. QSP models encode multiscale biology but are often under-identified with available data. Hybrid strategies can use ML to learn reduced-order representations, to map high-dimensional biomarkers onto a smaller set of latent states, or to estimate difficult parameters while respecting mechanistic constraints. The benefit is improved calibration to data while keeping causal interpretability for intervention simulations. Risks include embedding biases from training datasets and over-conferring confidence in unvalidated pathway components. Robust use requires transparent assumptions, sensitivity analysis, and validation against independent perturbations where feasible.

### 8.3 Regulatory relevance and future acceptance

Regulators value mechanistic transparency and have been cautious with black-box ML. Hybrid models reduce this tension by keeping a mechanistic core while using ML where it adds measurable value. Agencies are developing expectations for AI/ML in drug development, including documentation of training data, performance metrics, and change control (FDA, 2023b; FDA, 2022). For hybrid submissions, reviewers focus on fitness for purpose. Key questions: does the model extrapolate beyond observed data? Is uncertainty quantified? Are conclusions robust to alternative assumptions? Is the workflow reproducible? Clear reporting of model structure, parameterization, data provenance, and validation plans makes hybrid models easier to evaluate and therefore more likely to be accepted for decision support.

An important distinction exists between exploratory digital measures, which are used for hypothesis generation and internal modeling decisions, and decision-grade digital endpoints, which have undergone analytical validation, clinical validation, and defined context-of-use qualification per FDA guidance (FDA, 2023a). For example, actigraphy-derived rest–activity measures may support internal exposure–response modeling (exploratory), but would require formal biomarker qualification before supporting labeling claims or dose adjustment recommendations (decision-grade).

### 8.4 Emerging methodological advances

Physics-informed neural networks (PINNs) embed mechanistic laws, such as PK/PD differential equations, into the training objective of a neural network. This enforces pharmacological behavior while allowing flexible function approximation where data are informative. Nasim and Nasim (2024) introduced pharmacokinetic-informed neural networks that incorporate compartmental dynamics as constraints during learning (Nasim et al., 2024). In principle, this reduces data requirements and improves extrapolation compared with purely data-driven models. The practical questions for regulated use are identifiability, uncertainty quantification, and interpretability. A useful pattern is to treat PINNs as an alternative numerical representation of a mechanistic model, then apply familiar validation tools: sensitivity analyses, predictive checks, and external validation remain essential.

### 8.5 Practical guidance and best practices

The guidance in this section reflects the authors’ interpretation of the evidence reviewed above, combined with practical modeling experience. Where specific recommendations lack direct published support, they are framed as the authors’ perspective rather than established standards.

Several practical patterns consistently separate robust hybrid applications from fragile ones.

First, data quality and preprocessing remain foundational. Integrating preclinical, clinical, *in vitro*, and sensor outputs requires harmonized identifiers, units, time stamps, and metadata. Handling missingness, normalization, and bias mitigation should be treated as part of the modeling workflow, not as an afterthought (Zhang et al., 2022).

Second, define the mechanistic core first. It should capture the known causal structure and enforce physiological constraints. Add an ML component only where data support it, for residual structure, covariate effects, or measurement mappings.

Third, prioritize interpretability for the decisions the model will support. Feature importance or partial dependence analyses are starting points. Translate learned relationships into testable hypotheses or into covariate effects that can be stress-tested with simulations.

Fourth, invest in validation that matches the intended use. This includes posterior predictive checks, bootstrapping, and sensitivity to priors and preprocessing (internal), as well as cross-study, cross-site, cross-species, or cross-device validation (external) when feasible.

Fifth, document provenance, model versions, and training regimes. Reproducibility and auditability are increasingly expected in regulated contexts and make models easier to maintain as new data arrive.

## 9 Conclusion

Hybrid mechanistic–ML PK/PD modeling extends established pharmacometric practice by integrating data-driven components into a mechanistic framework when structural uncertainty, high-dimensional covariates, or complex nonlinear relationships limit purely mechanistic specification. The evidence reviewed here indicates that hybrid approaches can improve parameter estimation, covariate discovery, and translational scaling, particularly when dense digital biomarker time-series are available and the mechanistic model structure is sound.

Digital biomarkers strengthen translation when they capture physiology and behavior continuously and non-invasively. In preclinical studies, home-cage monitoring and telemetry provide dense measures of activity, temperature, sleep-like patterns, and autonomic signals. In clinical settings, wearables and remote monitoring provide analogous streams, enabling endpoint alignment across species. When linked to exposure with mechanistic structure, these signals can improve identification of delays, tolerance, and covariate effects, and can help reduce uncertainty in dose selection. The key is to define features that are biologically anchored and robust to device and site variation, rather than relying on opaque composite scores.

Credibility depends on validation, interpretability, and change control. Added ML flexibility can improve fit but can also introduce instability, leakage, and implausible extrapolation. Hybrid workflows should document preprocessing, training regimes, and model versions, and use diagnostics that test both the mechanistic and learned components. Where the ML component affects a key decision, uncertainty should be propagated into simulations and decision metrics.

Looking ahead, methods that couple mechanistic constraints with scalable learning are likely to expand, including PINN-like formulations, automated feature extraction from sensor data, and workflows for continual learning with strict governance. The most important differentiator for adoption in drug development will be fitness for purpose. Models that are transparent, reproducible, and prospectively validated will earn regulatory and scientific trust. Those that are merely complex will not. Progress depends less on algorithmic novelty and more on building the evidence base that connects model performance to decision quality in real drug development programs.
